# Astragaloside IV Alleviates Brain Injury Induced by Hypoxia via the Calpain-1 Signaling Pathway

**DOI:** 10.1155/2022/6509981

**Published:** 2022-12-03

**Authors:** Yan Meng, Shengxue Yu, Fang Zhao, Yu Liu, Yue Wang, Siqi Fan, Yuhong Su, Meili Lu, Hongxin Wang

**Affiliations:** ^1^Department of Liaoning Key Laboratory of Cardiovascular and Cerebrovascular Drugs, Jinzhou Medical University, Jinzhou 121000, China; ^2^Liaoning Key Laboratory of Diabetic Cognitive and Perceptive Dysfunction, Jinzhou 121000, China; ^3^College of Food and Health, Jinzhou Medical University, Jinzhou 121000, China

## Abstract

Long-term hypoxia can induce oxidative stress and apoptosis in hippocampal neurons that can lead to brain injury diseases. Astragaloside IV (AS-IV) is widely used in the antiapoptotic therapy of brain injury diseases. However, its mechanism of action is still not fully understood. In this study, we investigated the effect of AS-IV on hypoxia-induced oxidative stress and apoptosis in hippocampal neurons and explored its possible mechanism. In vivo, mice were placed in a hypoxic circulatory device containing 10% O_2_ and gavaged with AS-IV (60 and 120 mg/kg/d) for 4 weeks. In vitro, mouse hippocampal neuronal cells (HT22) were treated with hypoxia (1% O_2_) for 24 hours in the presence or absence of AS-IV, MDL-28170 (calpain-1 inhibitor), or YC-1 (HIF-1*α* inhibitor). The protective effect of AS-IV on brain injury was further explored by examining calpain-1 knockout mice. The results showed that hypoxia induced damage to hippocampal neurons, impaired spatial learning and memory abilities, and increased oxidative stress and apoptosis. Treatment with AS-IV or calpain-1 knockout improved the damage to hippocampal neurons and spatial learning and memory, attenuated oxidative stress and inhibited cell apoptosis. These changes were verified in HT22 cells. Overexpression of calpain-1 abolished the improvement of AS-IV on apoptosis and oxidative stress. In addition, the effects of AS-IV were accompanied by decreased calpain-1 and HIF-1*α* expression, and YC-1 showed a similar effect as AS-IV on calpain-1 and caspase-3 expression. In conclusion, this study demonstrates that AS-IV can downregulate the calpain-1/HIF-1*α*/caspase-3 pathway and inhibit oxidative stress and apoptosis of hippocampal neurons induced by hypoxia, which provides new ideas for studying the antiapoptotic activity of AS-IV.

## 1. Introduction

Brain injury has an estimated worldwide incidence of 1/500. Children under the age of 4, adults under the age of 30, and the elderly over 65 years of age are particularly severely affectedd [1]. Brain injury is one of the main causes of disability and death [2], and it has become a main factor affecting human health [3]. Hypoxia is one of the main contributors to brain injury. Hypoxia alters brain metabolism and function, which in turn leads to hippocampal nerve function injury [4]. Multiple mechanisms are involved in the development of hippocampal neuronal injury including oxidative stress and apoptosis [5].

Calpain is a conserved and specific neutral cysteine protease activated by Ca^2+^ [6]. Calpain-1 in the calpain family is the most commonly expressed subtype in the brain [7]; it forms a heterodimer with calpain small subunit-1 (calpain-4), which is necessary for the catalytic activity of the enzyme [8]. Since its discovery, calpain-1 has been associated with the pathological state of many diseases. Under hypoxic conditions, calpain-1 imbalance can lead to acute and chronic neurodegeneration [9], and calpain-1 imbalance will result in intracellular Ca^2+^ overload, which activates many Ca^2+^-dependent proteases [10] regulate the opening of mitochondrial permeability transition pore channels, release cytochrome c, specifically activate caspase-3, and participate in cytoskeleton remodeling, apoptosis, and necrosis [11, 12]. The role of calpain-1 in promoting apoptosis in brain diseases has been confirmed [13]. In addition, studies have shown that a potential mechanism of apoptosis is oxidative stress [14]. We speculate that calpain-1 is involved in oxidative stress and apoptosis in hypoxia induced brain injury.

Hypoxia inducible factor-1*α* (HIF-1*α*) is expressed in hypoxia and degraded in normoxia [15]. Studies have shown that inhibiting calpain-1 activity reduces the expression of HIF-1*α* and promotes the recovery of neural function [16]. In addition, HIF-1*α* plays a key role in oxidative stress in brain hypoxia models [17]. These studies suggest that HIF-1*α* may be involved in the mechanism by which calpain-1 promotes apoptosis. However, no studies have confirmed the mechanism of calpain-1/HIF-1*α* in hypoxia induced brain injury. We boldly speculate that calpain-1/HIF-1*α* protein pathway plays a key role in hypoxia induced oxidative stress and apoptosis.

Astragaloside IV (AS-IV) is a natural triterpene saponin extracted from Astragalus membranaceus [18]. AS-IV has many pharmacological activities and plays an important role in many diseases, such as antioxidant [19] and antiapoptosis [20], especially in preventing brain injury and protecting neurons; it can improve stroke angiogenesis through HIF-1*α* pathway [21]. However, the effect of AS-IV on hypoxia induced brain injury through calpain-1/HIF-1*α* pathway is still unclear. Therefore, this experiment analyzed the expression and distribution of the apoptotic proteins calpain-1, HIF-1*α*, Bax, Bcl-2, and caspase-3 in vivo and in vitro to determine whether AS-IV can improve hippocampal neuron injury through the calpain-1/HIF-1*α*/caspase-3 pathway and play antiapoptotic and antioxidative stress roles.

## 2. Materials and Methods

### 2.1. Materials and Reagents

AS-IV was obtained from Nanjing Jingzhu Biotechnology Company (purity>98% measured by HPLC, Nanjing. China). Assay kits for Cell Counting Kit-8 (CCK-8 kit), dihydroethidium (DHE), malondialdehyde (MDA), lactate (LD), blood urea nitrogen (BUN), lactate dehydrogenase (LDH), superoxide dismutase (SOD), glutathione peroxidase (GSH-Px), and JC-1 were purchased from Beyotime Biotechnology (Shanghai, China). A transferase-mediated dUTP nick-end labeling (TUNEL) kit was purchased from Roche (Darmstadt, Germany). Calpain-1 antibody (Cat No. 667732-1-Ig) was purchased from Proteintech (Wuhan, China). MDL-28170 (Cat No. M7934) and YC-1 (Cat No. M3991) were purchased from Abmole Bioscience (Houston, USA). Antibodies against Bcl-2 (A0208), Bax (A0207), Caspase-3 (A2156), and *β*-actin (AC038) were purchased from ABclonal (ABclonal Technology Co., Ltd.). Mouse hippocampal neuron cells (HT22) and HT22 cell-specific medium were purchased from Procell (Wuhan, China).

### 2.2. Animal Models and Drug Treatments

Sixty male wild-type C57BL/6 mice (Liaoning Changsheng Biotechnology Co., Ltd.) were randomly divided into the following 6 groups (*n* = 10 per group): control group (Con), hypoxia model group (HBD), HBD + AS-IV low-dose group (ASIV 60, 60 mg/kg/d), AS-IV high-dose group (ASIV 120, 120 mg/kg/d), MDL-28170 group (HBD + MDL, 20 mg/kg), and YC-1 group (HBD + YC-1, 20 mg/kg). Twenty male mice lacking *μ*-calpain (Capn1 EK684-/-) were randomly divided into the following two groups (*n* = 10 per group): knockout (CK) and hypoxia knockout (MK). Mice were exposed to 10% oxygen for 12 hours each day. Control mice were placed in cages containing normoxic gas under the same conditions. During hypoxia, the mice in the AS-IV group were given AS-IV at 60 mg/kg and 120 mg/kg per day. After 4 weeks, the mice were anesthetized with isoflurane and killed, and then the brain tissue was used for related pathological research.

### 2.3. Cell Experiments

HT22 cells were purchased from Wuhan Procelli and cultured in HT22 cell special medium (DMEM) containing 10% fetal bovine serum (FBS) and 1% penicillin streptomycin (P/s) at 37°C and a carbon dioxide concentration of 5%. AS-IV (50 *μ*mol/L and 100 *μ*mol/L), MDL-28170 (calpain-1 inhibitor; 20 *μ*mol/L), and YC-1 (HIF-1*α* inhibitor; 20 *μ*mol/L) were added to the cells 30 minutes before hypoxia, and hypoxia (1% oxygen) was applied for 24 hours. To further study the potential mechanism of AS-IV inhibiting calpain-1 in regulating hypoxia-induced brain injury, CAPN1-lentiviral vector (pLV-CAPN1, PRO-363, purchased from Genechem Co., LTD. Shanghai, China) was added to HT22 cells for 12 hours according to the manufacturer's instructions. Then, the transfected cells were exposed to hypoxia (1% oxygen) for 24 hours. The efficacy of overexpression was verified by western blot analysis.

### 2.4. Measurement of MDA, LD, and BUN Levels and LDH, SOD, and GSH-Px Activities

The homogenized brain tissue was centrifuged (3000 r/10 min), and the supernatant was collected to measure the MDA, LD, and BUN contents and SOD, LDH, and GSH-Px activities. HT22 cells were lysed; after which, SOD and GSH-Px activity and MDA content were detected according to the manufacturer's instructions and normalized to total protein content. All analyses were performed in accordance with the kits' requirements.

### 2.5. Morris Water Maze

The Morris water maze (MWM) test apparatus used was a circular tank equipped with a digital pick-up camera for monitoring animal behavior and a computer program for analyzing data (ZH0065, Zhenghua Bioequipment, Anhui, China). After modeling, 6 mice in each group were tested for MWM for 6 days. Each mouse explored two quadrants in the morning and the other two quadrants in the afternoon. On days 1-2, motor ability and visual conditions of the mice were detected by a visual platform experiment. On days 3-5, the escape latency and path length of the mice were detected by a hidden platform experiment. On day 6, mice passing through the target platform quadrant was detected by a hidden platform probe experiment.

### 2.6. HE Staining

Brain tissue was embedded in wax and cut into 4 *μ*m thick sections. The prepared slices were placed into an oven at 60°C, removed after drying for 90-120 min, placed into xylene for dewaxing, hydrated with graded ethanol, stained with HE, dehydrated with gradient ethanol, cleared with xylene, sealed with neutral gum, and used for microscope observation, photography, and analysis after complete drying and hardening.

### 2.7. TUNEL Assay

The slices were placed in a 60°C drying oven for 2 h and then subjected to routine stripping. The brain tissue slices were rinsed with PBS 3 times, 0.3% triaxone for 15 minutes, and 0.5% trypsin. The brain slices were incubated at 37°C for 15 minutes to rapidly degrade cell membrane proteins, and then the brain tissue sections were rinsed twice with PBS. Then, 50 *μ*L of TUNEL solution was added to each brain tissue section, incubated in an incubator at 37°C for 1 hour, and rinsed with PBS twice. POD was added dropwise and the section incubated in an incubator at 37°C for half an hour without light. Brain tissue sections were rinsed twice with PBS; DAB chromogenic solution was added dropwise for 30 s, and photos were taken under a laser confocal microscope after conventional dehydration, transparency, and sealing.

### 2.8. Immunofluorescence

The collected cells were fixed in 4% paraformaldehyde and washed three times with PBS. The sections were permeabilized with 0.3% Triton X-100 for 10 minutes and then incubated with 5% bovine serum albumin for 30 minutes. The sections were incubated with primary antibody against anti-calpain-1 (1: 100) at 4°C overnight. The next day, the cells were stained with the corresponding secondary antibody and DAPI. Image analysis was performed using Image-Pro Plus software.

### 2.9. Western Blot

The collected brain tissue and HT22 cells were homogenized in ice-cold RIPA lysis buffer. The protein concentration was determined using a BCA protein assay kit. The samples were separated by SDS–PAGE (8%-12% polyacrylamide gel) and then transferred to PVDF membranes, which were blocked with 1% BSA for 1.5 hours and then incubated with antibodies against calpain-1, caspase-3, Bax, Bcl-2, GAPDH, and *β*-actin overnight at 4°C. The next day, the membranes were washed 3 times with TBST and then incubated with an HRP-conjugated secondary antibody at room temperature for 1 hour. The membrane was visualized using an ECL detection kit. The results were analyzed with ImageJ software.

### 2.10. Cell Viability Assay

A CCK-8 Kit was used to detect the viability of HT22 cells under different conditions. Cell viability was calculated based on the percentage of optical density relative to the untreated control group.

### 2.11. Detection of Mitochondrial Membrane Potential

The mitochondrial membrane potential of HT22 cells was detected by JC-1 fluorescence staining according to the manufacturer's instructions and our previous report [22]. Briefly, the cells were incubated with JC-1 at 10 *μ*g/ml for 15 min at 37°C, and then images were observed by fluorescence microscopy. Increased JC-1 (green) fluorescence levels and decreased JC-1 (red) fluorescence levels indicated mitochondrial membrane potential depolarization. All images were analyzed by ImageJ software.

### 2.12. Measurement of Reactive Oxygen Species Production

Intracellular ROS were detected by DHE staining, and ROS were assessed in HT22 cells and the hippocampal CA1 region according to the manufacturer's instructions. The images were captured using fluorescence microscopy and analyzed using ImageJ software according to our previous report [22].

### 2.13. Immunohistochemistry

Immunohistochemical staining was used to detect the levels of calpain-1 and caspase-3 in hippocampal CA1 area. The collected hippocampal CA1 area of brain tissue was fixed with 4% paraformaldehyde overnight, embedded in paraffin, and sliced. After dewaxing and antigen recovery, the slices were diluted with primary antibodies against calpain-1 and caspase-3 at a ratio of 1 : 100 and incubated overnight at 4°C. After washing with PBS, that second antibody combined with horseradish peroxidase was incubated at 37°C for 20 min, added 50 *μ*l DAB solution to the hippocampal CA1 section of each brain tissue, and stained for 1-3 min. After washing with running water, hematoxylin was redyed for 5 min at room temperature and was observed and captured with slice Leica DMI 3000B microscope. ImageJ software was used to calculate the percentage of positive cells.

### 2.14. Data Analysis

All data are expressed as the mean ± SD of normally distributed data. One-way ANOVA or repeated measures ANOVA were used for data testing. All statistical analyses were performed using SPSS 26.0 software. *P* < 0.05 was considered statistically significant.

## 3. Results

### 3.1. AS-IV and Calpain-1 Knockout Protect against Hippocampal Neurons in Hypoxia-Induced Brain Injury in Mice

**Figure 1:**
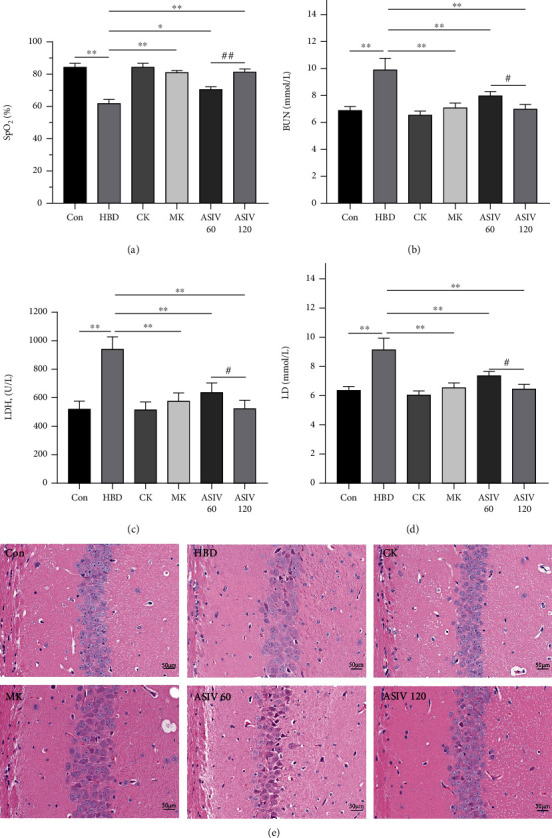
AS-IV and calpain-1 knockout improve hypoxia-induced hippocampal neuronal injury. (a) SpO_2_ of mice in each group was detected by a blood gas analyzer (*n* = 4; *F*_(5, 17)_ = 62.149). (b–d) BUN, LDH, and LD determination kit was used to detect the changes in BUN, LDH, and LD, respectively (*n* = 4; BUN, *F*_(5, 17)_ = 27.023; LDH, *F*_(5, 17)_ = 21.551; LD, *F*_(5, 17)_ = 14.832). (e) HE staining was used to detect pathological changes in the hippocampal CA1 area (*n* = 3). Data are presented as the mean ± SD. ^∗^*P* < 0.05, ^∗∗^*P* < 0.01 compared with the HBD group, ^#^*P* < 0.05, ^##^*P* < 0.01 compared with the ASIV 60 mg/kg group.

To study the effects of AS-IV and calpain-1 knockout on hippocampal neurons in mice with hypoxic brain injury, we detected the relevant biochemical indices. Figures 1(a)–1(e) show that compared with the control group, SpO_2_ decreased; LDH, BUN, and LD increased. In HBD group, the cell boundary of hippocampal CA1 area was fuzzy and disordered. Compared with the HBD group, calpain-1 knockout and AS-IV reversed these changes. These results showed that AS-IV and calpain-1 gene knockout had protective effects on hippocampal neuron injury in mice with hypoxic brain injury. Compared with 60 mg/kg, 120 mg/kg AS-IV has better improvement effect.

### 3.2. AS-IV and Calpain-1 Knockout Improve Spatial Learning and Memory in Mice with Hypoxic Brain Injury

**Figure 2:**
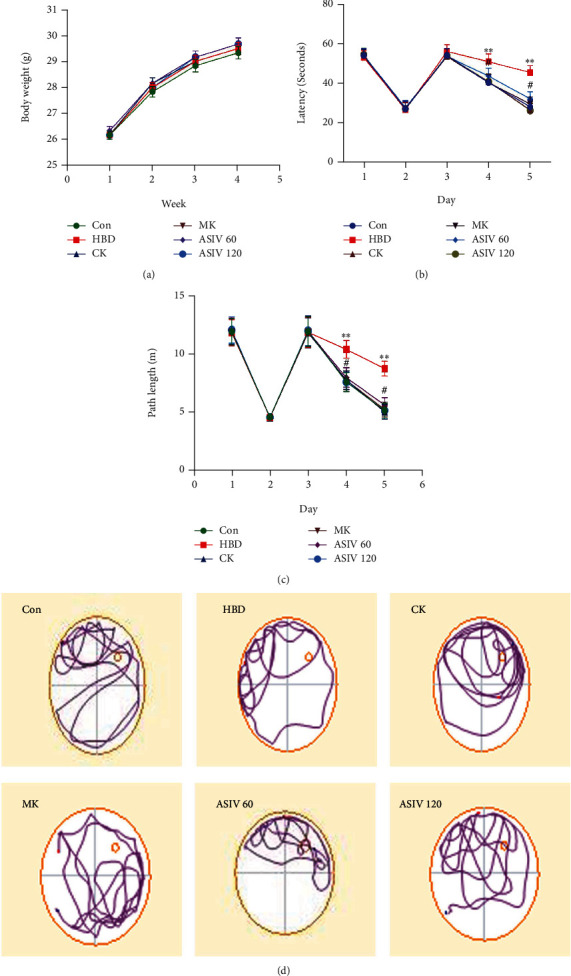
Effects of AS-IV and calpain-1 knockout on spatial learning and memory in mice with hypoxic brain injury. (a) The weight difference of mice in each group was detected by a weight scale (*n* = 6; *F*_(5, 30)_ = 0.209). (b–d) The Morris water maze test was used to detect escape latency, path length, and times of passing through the target quadrant (*n* = 6; latency, *F*_(5, 30)_ = 3.952; path length, *F*_(5, 30)_ = 4.515). Data are presented as the mean ± SD. ^∗∗^*P* < 0.01 compared with the HBD group, ^#^*P* < 0.05 compared with the ASIV 60 mg/kg group.

The Morris water maze experiment was used to observe the effects of AS-IV and calpain-1 gene knockout on learning and memory in mice with hypoxic brain injury. There was no significant difference in body weight between the groups during hypoxia (Figure 2(a)). In the visual platform test (2 days), there was no significant difference in escape latency or path length, which showed that hypoxia and administration did not significantly change the movement or vision of mice. In the hidden platform experiment (3 days), the escape latency and path length of mice in the HBD group were longer than those in the control group (Figures 2(b) and 2(c)). In addition, the covert platform probe test (1 day) showed that mice in the HBD group passed through the target quadrant less than those in the control group (Figure 2(d)), and calpain-1 knockout and AS-IV could reverse these changes in mice in the HBD group. This finding suggests that calpain-1 knockout and AS-IV can improve the learning and memory abilities of mice with hypoxic brain injury.

### 3.3. AS-IV and Calpain-1 Knockout Improve Oxidative Stress in Mice with Hypoxic Brain Injury

**Figure 3:**
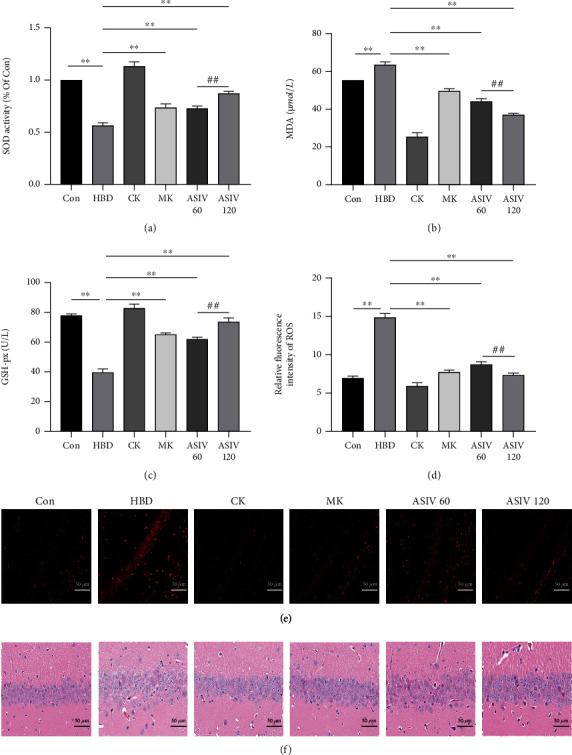
AS-IV and calpain-1 knockout can reduce the oxidative stress of hippocampal CA1 neurons in mice with hypoxic brain injury. (a–c) The changes in SOD, MDA, and GSH-Px were measured by SOD, MDA, and GSH-Px determination kits, respectively (*n* = 4; SOD, *F*_(5, 17)_ = 170.353; MDA, *F*_(5, 17)_ = 280.493; GSH-Px, *F*_(5, 17)_ = 201.152). (d–e) The DHE probe labeling method was used to observe the production of reactive oxygen species in the hippocampal CA1 region of mice in each group (*n* = 3; *F*_(5, 12)_ = 255.003). (f) Morphological changes of hippocampal CA1 neurons observed by HE staining (*n* = 3). Data are presented as the mean ± SD. ^∗∗^*P* < 0.01 compared with the HBD group, ^##^*P* < 0.01 compared with the ASIV 60 mg/kg group.

To study the effects of AS-IV and calpain-1 knockout on oxidative stress in mice with hypoxic brain injury, SOD, MDA, and GSH-Px assay kits were used in the current study. The DHE probe labeling method and HE staining were performed to observe the changes in various indices. As shown in Figures 3(a)–3(c), compared with the control group, in the HBD group, SOD activity and GSH-Px contents decreased significantly, MDA contents increased significantly, ROS generation in the hippocampal CA1 region increased (Figures 3(d) and 3(e)), and cell boundaries were blurred and disordered (Figure 3(f)), while calpain-1 knockout and AS-IV reversed these changes. Our results show that AS-IV and calpain-1 knockout can improve oxidative stress in mice with hypoxic brain injury.

### 3.4. AS-IV and Calpain-1 Knockout Attenuate Apoptosis in Mice with Hypoxic Brain Injury

**Figure 4:**
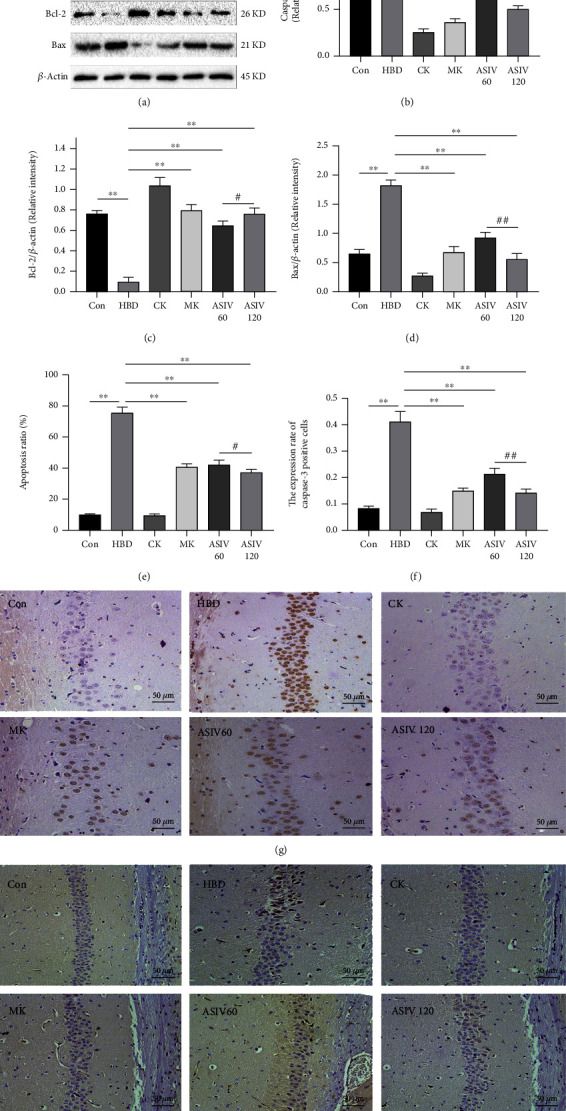
AS-IV and calpain-1 knockout reduced the apoptosis of hippocampal neurons in mice with hypoxic brain injury. (a–d) Caspase-3, Bax, and Bcl-2 protein expression levels were observed by Western blotting (n = 3; Caspase-3, *F*_(5, 12)_ = 200.389; Bax, *F*_(5, 12)_ = 117.226; Bcl-2, *F*_(5, 12)_ = 75.166). (e, g) TUNEL staining was used to observe the number and percentage of TUNEL-positive cells in the hippocampal CA1 area of mice in each group (n = 3; *F*_(5, 12)_ = 373.068). (f, h) Immunohistochemistry was used to observe caspase-3 protein expression in the CA1 region of the mouse hippocampus (*n* = 3; *F*_(5, 12)_ = 124.563). Data are presented as the mean ± SD. ^∗∗^*P* < 0.01 compared with the HBD group, ^#^*P* < 0.05, ^##^*P* < 0.01 compared with the ASIV 60 mg/kg group.

AS-IV cannot only attenuate oxidative stress in hypoxic brain injury but also attenuate apoptosis in hypoxic brain injury in mice. Western blotting, TUNEL staining, and immunohistochemistry were used to observe the apoptosis of mice in each group. The results showed that compared with the control group, in the HBD group, caspase-3 and Bax protein expression increased, Bcl-2 protein expression decreased (Figures 4(a)–4(d)), the number of TUNEL-positive cells increased (Figures 4(e) and 4(g)), and caspase-3 expression increased in the hippocampal CA1 area (Figures 4(f) and 4(h)). Compared with the HBD group, calpain-1 knockout and AS-IV reversed these apoptotic levels. The above results showed that AS-IV and calpain-1 knockout could significantly reduce apoptosis in mice with hypoxic brain injury, and 120 mg/kg AS-IV has more obvious inhibitory effect than 60 mg/kg AS-IV.

### 3.5. AS-IV and Calpain-1 Knockout Inhibit Hypoxia-Induced Activation of the Calpain-1/HIF-1*α*/Caspase-3 Pathway in Mouse Hippocampal Neurons

**Figure 5:**
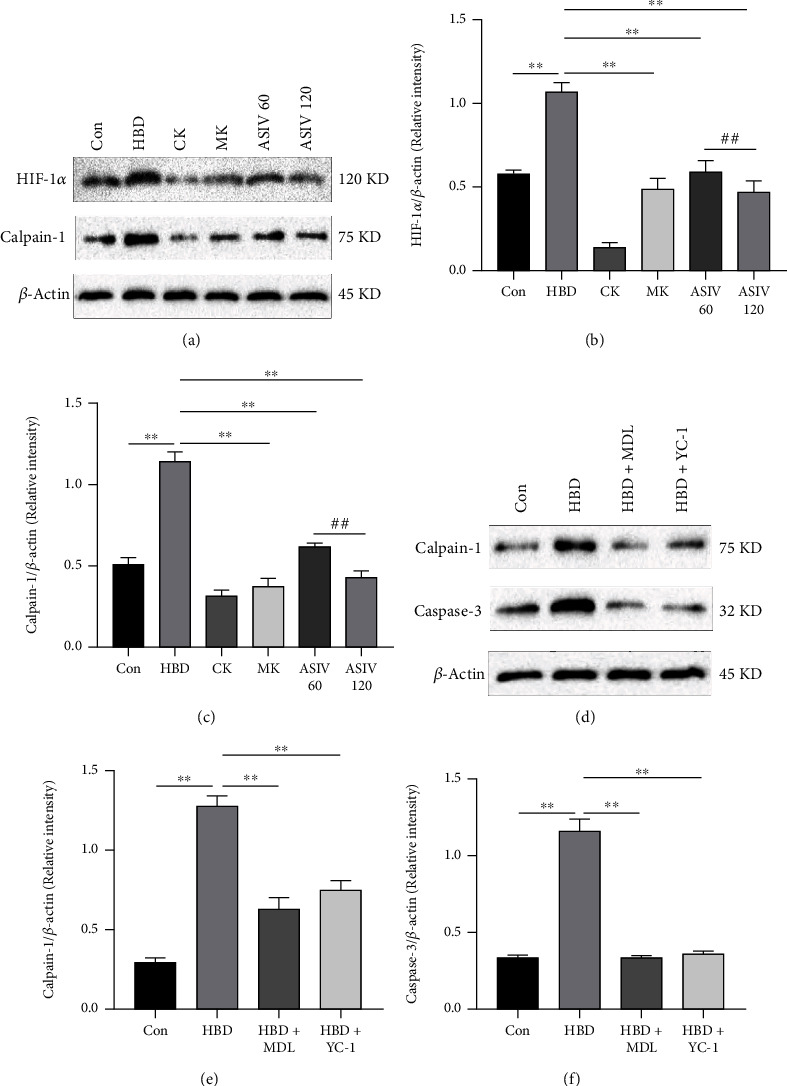
AS-IV and calpain-1 knockout inhibit hypoxia-induced activation of the calpain-1/HIF-1*α*/caspase-3 pathway in mouse hippocampal neurons. (a–c) Western blotting was used to detect calpain-1 and HIF-1*α* protein expression (*n* = 3; calpain-1, *F*_(5, 12)_ = 201.609; HIF-1*α*, *F*_(5, 12)_ = 103.832). (d–f) After YC-1 was introduced, the protein expression of calpain-1 and caspase-3 was detected by Western blotting (*n* = 3; calpain-1, *F*_(3, 8)_ = 180.664; caspase-3, *F*_(3, 8)_ = 351.612). Data are presented as the mean ± SD. ^∗∗^*P* < 0.01 compared with the HBD group, ^##^*P* < 0.01 compared with the ASIV 60 mg/kg group.

To demonstrate that AS-IV and calpain-1 gene knockout inhibit hypoxia-induced activation of the calpain-1/HIF-1*α*/caspase-3 pathway in mouse hippocampal neurons, Western blotting and immunohistochemistry were used to detect the protein expression levels of calpain-1, HIF-1*α* and caspase-3. Compared with that in the control group, calpain-1 and HIF-1*α* protein expression was increased in the HBD group (Figures 5(a)–5(c)). Compared with the HBD group, calpain-1 knockout and AS-IV could reverse these changes, suggesting that calpain-1 knockout and AS-IV could inhibit the activation of the calpain-1/HIF-1*α* pathway in hippocampal neurons. To further confirm the pathway relationship, after introducing the HIF-1*α* inhibitor YC-1 to inhibit HIF-1*α*, the expression of calpain-1 and caspase-3 decreased (Figures 5(d)–5(f)). The above results showed that calpain-1 knockout and AS-IV inhibited the expression of calpain-1 in mice with hypoxic brain injury, and calpain-1 had positive feedback regulation with caspase-3 and HIF-1*α*.

### 3.6. AS-IV Improves Hypoxia-Induced HT22 Cell Injury and Oxidative Stress

**Figure 6:**
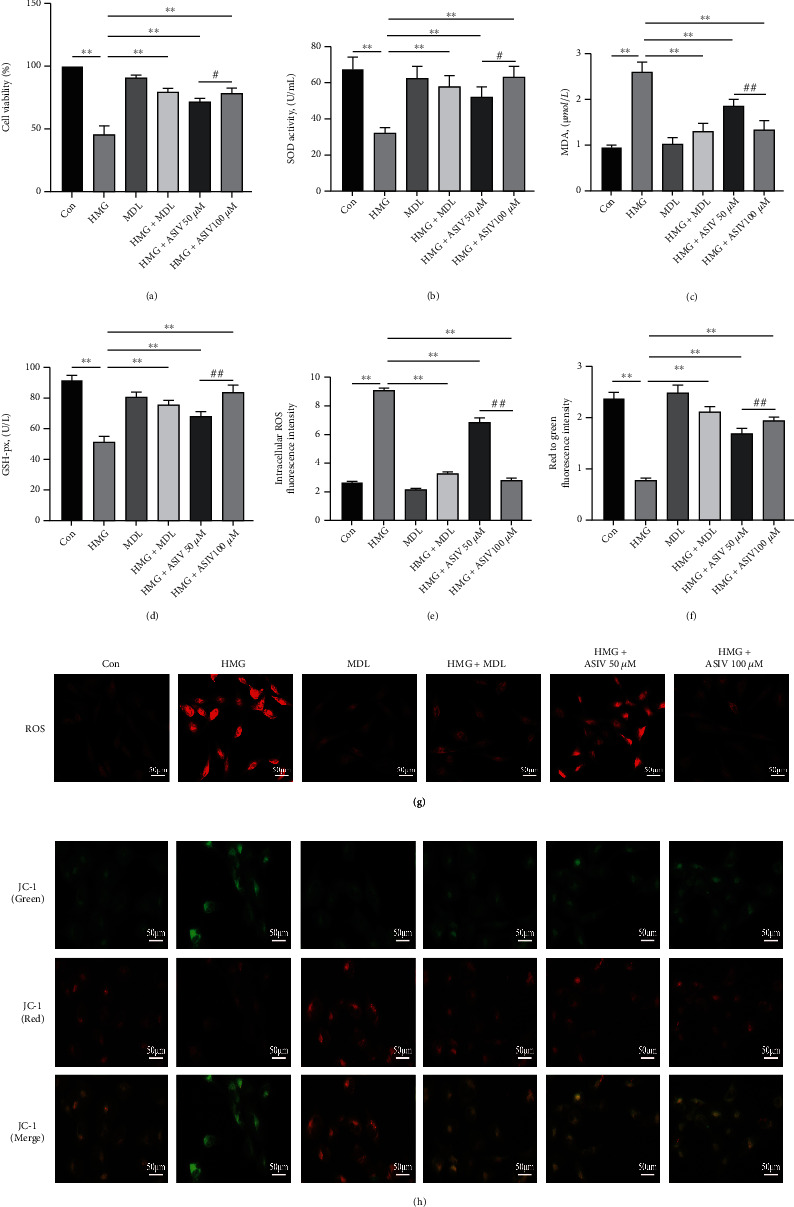
AS-IV improves hypoxia-induced HT22 cell injury and oxidative stress. (a) The cell viability of each group was detected by CCK-8 assay (*n* = 4; *F*_(5, 17)_ = 86.359). (b–d) Changes in SOD, MDA, and GSH-Px were detected with kits (*n* = 4; SOD, *F*_(5, 17)_ = 15.852; MDA, *F*_(5, 17)_ = 36.000; GSH-Px, *F*_(5, 17)_ = 56.030). (e, g) ROS fluorescence probe was used to observe the expression level of ROS (*n* = 3; *F*_(5, 12)_ = 1162.936). (f, h) The changes in the mitochondrial membrane potential of HT22 cells were detected by JC-1 (*n* = 3; *F*_(5, 12)_ = 119.470). Data are presented as the mean ± SD. ^∗∗^*P* < 0.01 compared with the HMG group, ^#^*P* < 0.05, ^##^*P* < 0.01 compared with the ASIV 50 *μ*M group.

Previously, we preliminarily confirmed the protective effect of AS-IV on hippocampal neurons and the improvement of oxidative stress in mice with hypoxic brain injury. We further verified this effect at the cellular level by CCK-8 assay; SOD, MDA, and GSH-Px determination kits; DHE probe labeling and mitochondrial membrane potential detection kits. The results showed that compared with the control group, in the HMG group, HT22 cell viability decreased (Figure 6(a)), SOD and GSH-Px activities decreased, MDA contents increased (Figures 6(b)–6(d)), ROS levels increased significantly (Figures 6(e) and 6(g)), and the mitochondrial membrane potential was abnormal (Figures 6(f) and 6(h)). Compared with HMG treatment, AS-IV treatment reversed these changes. The above results show that AS-IV has a protective effect on hypoxia-induced HT22 cell injury and can attenuate hypoxia-induced oxidative stress, similar to MDL-28170.

### 3.7. AS-IV Attenuates Hypoxia-Induced Apoptosis in HT22 Cells

**Figure 7:**
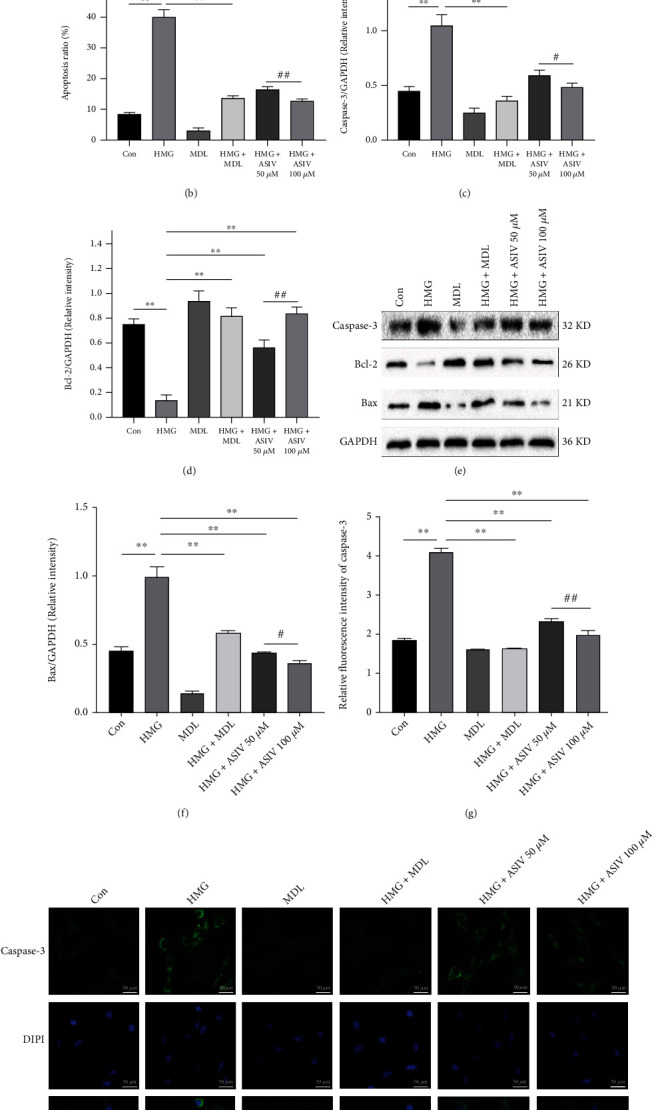
AS-IV inhibits apoptosis of hypoxic HT22 cells. (a, b) The apoptosis rate of HT22 cells was detected by flow cytometry (*n* = 3; *F*_(5, 12)_ = 343.883). (c–f) Caspase-3, Bax, and Bcl-2 protein expression levels were observed by Western blotting (*n* = 3; Caspase-3, *F*_(5, 12)_ = 75.979; Bax, *F*_(5, 12)_ = 170.087; Bcl-2, *F*_(5, 12)_ = 77.832). (g, h) The fluorescence expression of caspase-3 protein was observed by immunofluorescence (*n* = 3; *F*_(5, 12)_ = 713.355). Data are presented as the mean ± SD. ^∗∗^*P* < 0.01 compared with the HMG group, ^#^*P* < 0.05, ^##^*P* < 0.01 compared with the ASIV 50 *μ*M group.

To further prove the inhibitory effect of AS-IV on apoptosis, we detected apoptosis-related indices by flow cytometry, Western blotting, and immunofluorescence. Compared with the control group, in the HMG group, the number of positive cells and the apoptotic rate increased (Figures 7(a) and 7(b)), caspase-3 and Bax protein expression increased, Bcl-2 protein expression decreased Figures 7(c)–7(f)), and caspase-3 fluorescence expression increased (Figures 7(g) and 7(h)). Compared with the HMG group, AS-IV significantly reversed these changes. The results showed that AS-IV could inhibit the apoptosis of hypoxic HT22 cells, similar to MDL-28170.

### 3.8. AS-IV Inhibits Hypoxia-Induced Activation of the Calpain-1/HIF-1*α*/Caspase-3 Pathway in HT22 Cells

**Figure 8:**
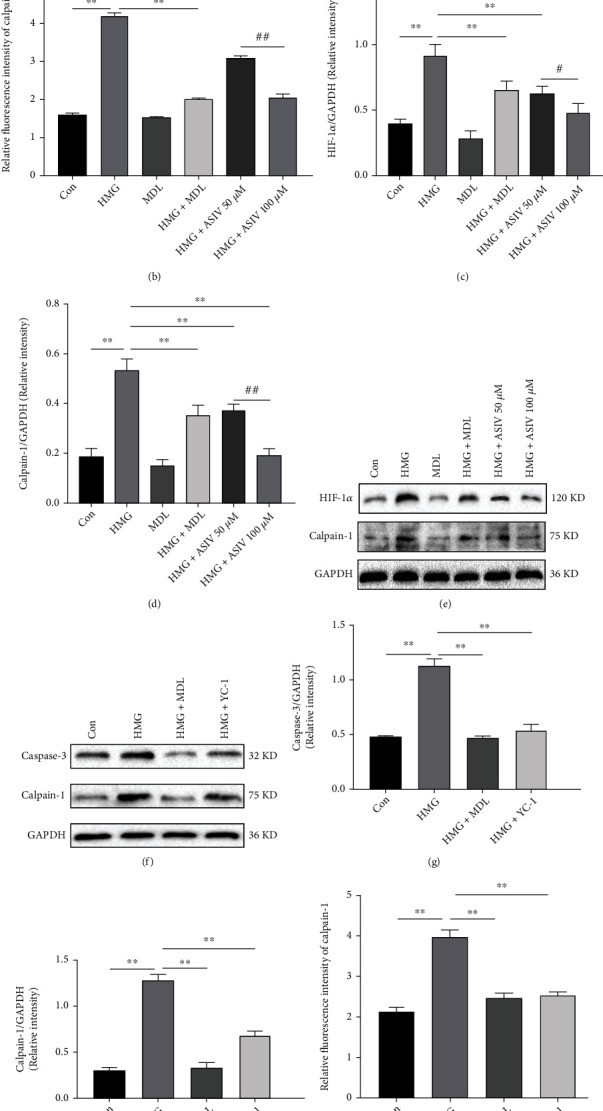
AS-IV inhibits hypoxia-induced activation of the calpain-1/HIF-1*α*/caspase-3 pathway in HT22 cells. (a, b) Calpain-1 expression in HT22 cells was observed by immunofluorescence (*n* = 3; *F*_(5, 12)_ = 855.217). (c–e) Western blotting was used to detect calpain-1 and HIF-1*α* protein expression (*n* = 3; calpain-1, *F*_(5, 12)_ = 52.836; HIF-1*α*, *F*_(5, 12)_ = 32.445). (f–h) After YC-1 was introduced, the protein expression of calpain-1 and caspase-3 was detected by Western blotting (*n* = 3; calpain-1, *F*_(3, 8)_ = 180.664; caspase-3, *F*_(3, 8)_ = 148.465). (i, j) After introducing YC-1, the expression of calpain-1 in HT22 cells was observed by immunofluorescence (*n* = 3; *F*_(3, 8)_ = 129.190). Data are presented as the mean ± SD. ^∗∗^*P* < 0.01 compared with the HMG group, ^#^*P* < 0.05, ^##^*P* < 0.01 compared with the ASIV 50 *μ*M group.

Previously, we preliminarily confirmed that AS-IV inhibits the expression of calpain-1 and the relationship between calpain-1 and HIF-1*α* at the animal level. Next, we further verified this finding at the cellular level by immunofluorescence and Western blotting. Compared with the control group, in the HMG group, calpain-1 and HIF-1*α* protein levels increased and calpain-1 fluorescence expression increased (Figures 8(a)–8(e)). Compared with HMG treatment, AS-IV treatment reversed the expression of calpain-1 and HIF-1*α*. In addition, calpain-1 and caspase-3 expression decreased significantly after treatment with YC-1 (Figures 8(f)–8(j)). Our results showed that AS-IV inhibited the expression of calpain-1 in HT22 cells induced by hypoxia, and calpain-1 was related to caspase-3 and HIF-1*α* through positive feedback.

### 3.9. The Effect of Calpain-1 Overexpression on AS-IV Improving Oxidative Stress and Apoptosis of HT22 Cells Induced by Hypoxia

**Figure 9:**
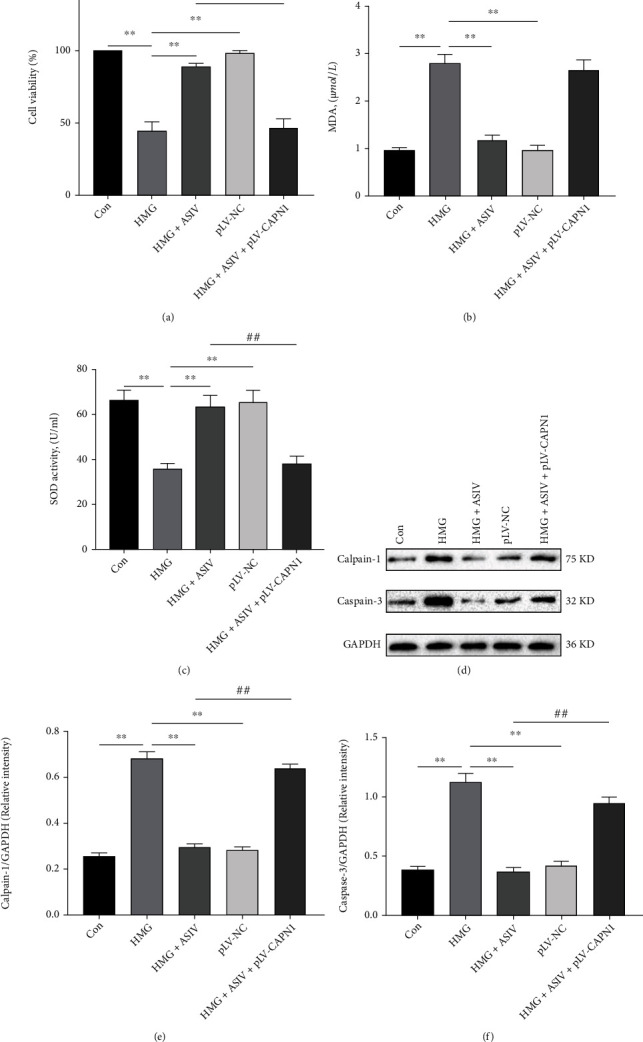
AS-IV inhibits calpain-1 overexpression and attenuates hypoxia-induced oxidative stress and apoptosis in HT22 cells. (a) The cell viability of each group was detected by CCK-8 assay (*n* = 4; *F*_(4, 15)_ = 126.041). (b, c) Changes in SOD and MDA were detected with kits (*n* = 4; SOD, *F*_(4, 15)_ = 38.705; MDA, *F*_(4, 15)_ = 102.154). (d–f) Western blotting was used to detect caspase-3 and calpain-1 protein expression (*n* = 3; calpain-1, *F*_(4, 10)_ = 342.774; caspase-3, *F*_(4, 10)_ = 160.375). Data are presented as the mean ± SD. ^∗∗^*P* < 0.01 compared with the HMG group, ^##^*P* < 0.01 compared with the HMG + ASIV group.

To further determine the role of AS-IV inhibiting calpain-1 in hypoxia-induced oxidative stress and apoptosis in HT22 cells, we introduced pLV-CAPN1 to overexpress calpain-1. Compared with HMG group, HMG + ASIV group increased the viability of hypoxia-induced HT22 cells (Figure 9(a)), decreased MDA, and increased SOD level (Figures 9(b) and 9(c)). In addition, AS-IV downregulated the expression level of caspase-3 and calpain-1 protein in hypoxia-induced HT22 cells (Figures 9(d)–9(f)). Compared with HMG + ASIV group, HMG + ASIV+pLV-CAPV1 group can reverse these changes. Our results further indicate that AS-IV can reduce oxidative stress and apoptosis of HT22 cells induced by hypoxia by inhibiting calpain-1 protein pathway.

## 4. Discussion

Hippocampal neurons are important functional units of the brain that are present in the hippocampus inside the temporal lobe of the brain. Lesions of hippocampal neurons are the main cause of brain injury-related diseases [23], and hippocampal neurons are also the first sites of brain injury. Therefore, hippocampal neurons play an important role in maintaining brain function and memory. Hypoxia-induced oxidative stress and apoptosis are considered to be the main factors of hippocampal neuron injury and an increased risk of brain diseases [24]. At present, mild hypothermia therapy is often used to treat brain injury induced by hypoxia. However, mild hypothermia therapy cannot completely protect the damaged brain tissue, nor can it effectively reduce the death risk and disability rate of hypoxic brain injury [25]. Therefore, exploring the specific mechanism of drugs on hypoxia-induced oxidative stress and apoptosis is the key to improve brain injury. Calpain-1 plays an important role in regulating nervous system diseases [26]. AS-IV showed neuroprotective effect in the study of brain injury [27]. The main findings of this study are that AS-IV can inhibit the expression of calpain-1 and caspase-3, inhibit ROS and reduce oxidative stress and apoptosis in vivo and in vitro, and improve brain injury after hypoxia. In addition, we found that calpain-1/HIF-1*α*/caspase-3 signaling pathway plays a key role in AS-IV in improving hypoxia-induced brain injury.

Oxidative stress refers to a state in which the body's oxidative and antioxidant defense systems are imbalanced, with the organism tending to an oxidative state, and is considered an important contributor to disease [28]. There is a close relationship between brain injury and oxidative stress. Oxidative stress has been found to be very active in hippocampal neurons and is an important contributor to hippocampal neuronal damage [29]. Mitochondria are important targets of oxidative stress, and brain hypoxia leads to mitochondrial oxidative phosphorylation overload [30], stimulates the excessive production of ROS [31], damages mitochondrial function, and causes oxidative stress damage to neurons [32]. In addition, cerebral hypoxia leads to disordered mitochondrial oxidative phosphorylation, a reduction in ATP production, and an increase in Ca^2+^ influx, which subsequently activate calpain. Calpains are thiol or cysteine proteases present in most mammalian cells [33]. Calpain-1, the major calpain family isoform, plays an important role in hypoxia-induced central system diseases [34], and studies have shown that calpain-1 plays an important role in oxidative stress injury [35]. Therefore, reducing the activity of calpain-1 is a good strategy in hypoxia-induced oxidative stress-related diseases. In our study, we examined the expression of calpain-1 and the effect of calpain-1 on oxidative stress both in vivo and in vitro. The results showed that calpain-1 knockout and AS-IV improved hypoxia-induced hippocampal neuronal damage and memory ability, inhibited the release and expression of calpain-1, reduced ROS production, and inhibited oxidative stress, and the high dose of AS-IV is better.

Apoptosis is a kind of programmed cell death found in animals [36], and it is the main mechanism of brain tissue injury after hypoxia. There are many factors through which hypoxia induces apoptosis, among which excessive activation of oxidative stress is a powerful factor to trigger apoptosis. Some studies have found that oxidative stress is an important factor leading to apoptosis of hippocampal neurons [37]. Hypoxia can activate oxidative stress to induce calcium release, which in turn activates calpain-1. Increased cytosolic Ca^2+^ may cause uncoupling of mitochondrial oxidative phosphorylation, inducing the mitochondrial membrane permeability transition (MPT) state, where Bcl-2 located on the mitochondrial membrane is inhibited, regulating the opening of the mitochondrial permeability transition pore (MPTP) channel, and thereby releasing cytochrome c [38]. Once cytochrome c is released from mitochondria, it specifically activates Caspase-3, which triggers a biochemical cascade [39]. Calpain has been implicated as a key protease during oxidative stress, and calpain-1 has been implicated in neuronal apoptosis [40, 41]. Our results showed that AS-IV improved hypoxia-induced calpain-1 release and expression, inhibited the expression of the apoptotic proteins caspase-3 and Bax, and upregulated Bcl-2 in hippocampal neurons. To further validate the role of calpain-1, we examined calpain-1 knockout mice. The results showed that calpain-1 knockout significantly inhibited the expression of the apoptotic proteins caspase-3 and Bax and upregulated Bcl-2 expression. Our data indicated that AS-IV could ameliorate hypoxia-induced apoptosis in hippocampal neurons via calpain-1; the high dose of AS-IV is better, and that calpain-1 played an important role in apoptosis.

Calpain-1-mediated hypoxia-induced oxidative stress and apoptosis involve a variety of protein molecules, among which hypoxia inducible factor-1*α* (HIF-1*α*) may play a crucial role. Studies have shown that calpain-1 is involved in many physiological and pathological phenomena [42], including the disruption of HIF-1*α* by hypoxia [43], and calpain-1 activation accelerates HIF-1*α* signal accumulation [44]. Therefore, we investigated the effect of AS-IV on the inhibition of the calpain-1/HIF-1*α* pathway in hypoxia-induced oxidative stress. Our results showed that calpain-1 knockdown or inhibition and AS-IV reduced hypoxia-induced calpain-1 and HIF-1*α* protein expression and reduced ROS generation in hippocampal neurons. In addition, some studies have shown that in a hypoxic brain injury model, hypoxia significantly induces HIF-1*α*, caspase-3, and cytochrome c expression, which promotes apoptosis and the development of brain injury [45]. This finding indicates that HIF-1*α* plays an important role in the process of regulating apoptosis. HIF-1*α* was shown to regulate MPTP channel opening and cytochrome c release and activate caspase-3 to trigger the apoptotic response. Therefore, we studied the effect of AS-IV on the inhibition of the calpain-1/HIF-1*α* pathway in hypoxia-induced apoptosis. Our results showed that calpain-1 knockdown or inhibition and AS-IV reduced hypoxia-induced calpain-1, HIF-1*α*, and caspase-3 protein expression in hippocampal neurons and reduced the number of apoptosis-positive cells as well as the apoptotic rate. Moreover, to further verify the pathway impact, we introduced YC-1 to inhibit HIF-1*α*, which also reversed the increase in calpain-1 and caspase-3 in hypoxic brain injury and significantly reduced the promotion of caspase-3 by calpain-1. This result suggested that AS-IV might ameliorate hypoxia-induced brain injury by inhibiting calpain-1/HIF-1*α*/caspase-3. To further explore the mechanism of AS-IV inhibiting calpain-1, we introduced pLV-CAPN1 to overexpress calpain-1. The results showed that AS-IV could inhibit the expression of caspase-3 and calpain-1, improve cell viability, reduce MDA content, increase SOD activity. The pLV-CAPN1 introduced can reverse these changes. The results showed that AS-IV inhibited calpain-1 protein pathway and improved oxidative stress and apoptosis induced by hypoxia.

In conclusion, the present study demonstrated a protective effect of AS-IV on hypoxia-induced brain injury. The improvement of AS-IV on brain injury may be achieved by inhibiting oxidative stress and apoptosis of hippocampal neurons via downregulation of the calpain-1/HIF-1*α*/caspase-3 signaling pathway. This not only indicates that calpain is expected to become an important target for drug treatment of brain injury, but also provides a new important idea for the clinical prevention and treatment of AS-IV as brain injury. In order to reduce the contingency of the experiment, ensure that the cause of a certain symptom during the experiment is objective, only male mice were used in current study. However, in order to better explore the protective effect of AS-IV on brain injury, future relevant research needs to be conducted at the level of both female and male mice.

## Data Availability

The data used to support the findings of this study are available from the corresponding author upon request.
